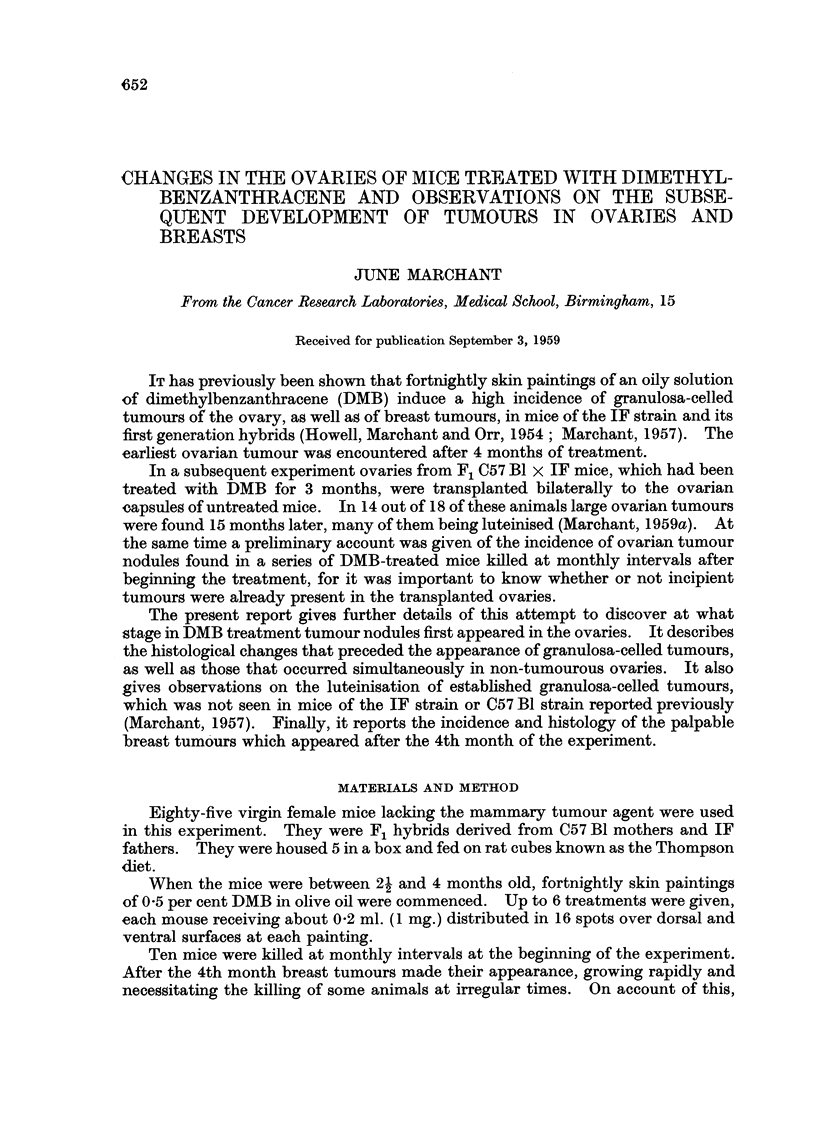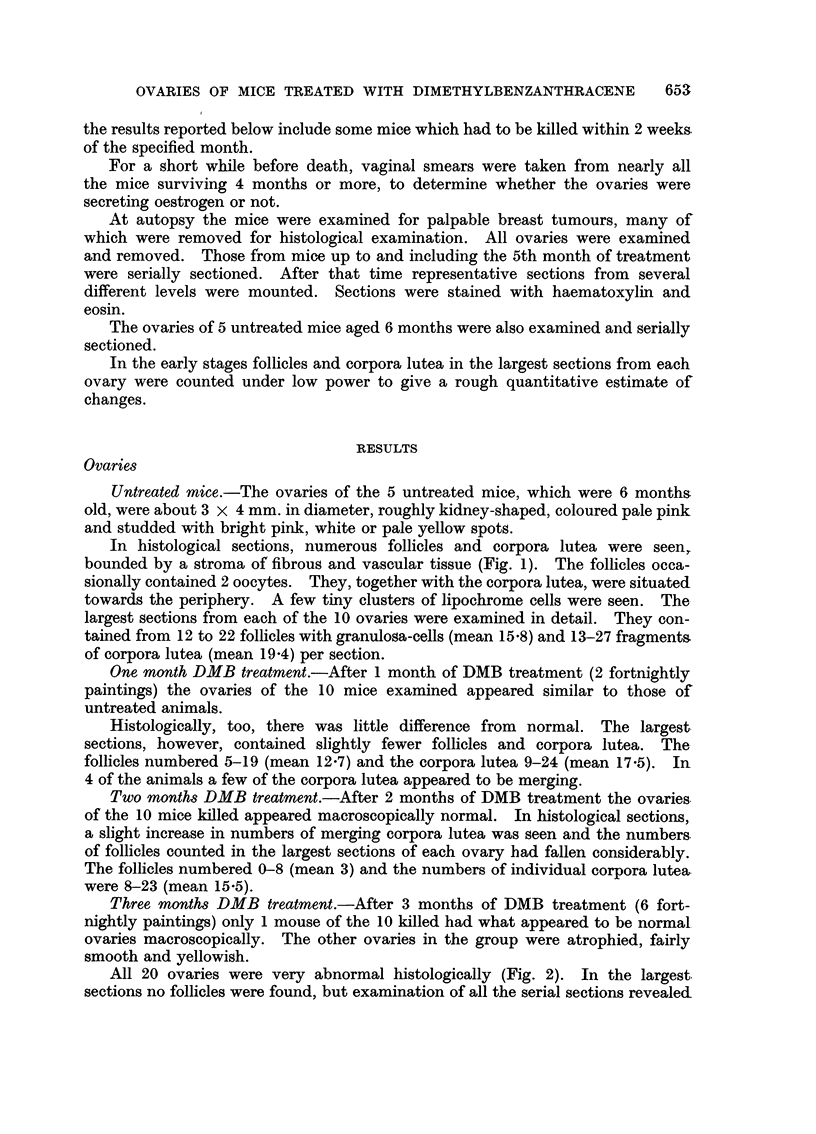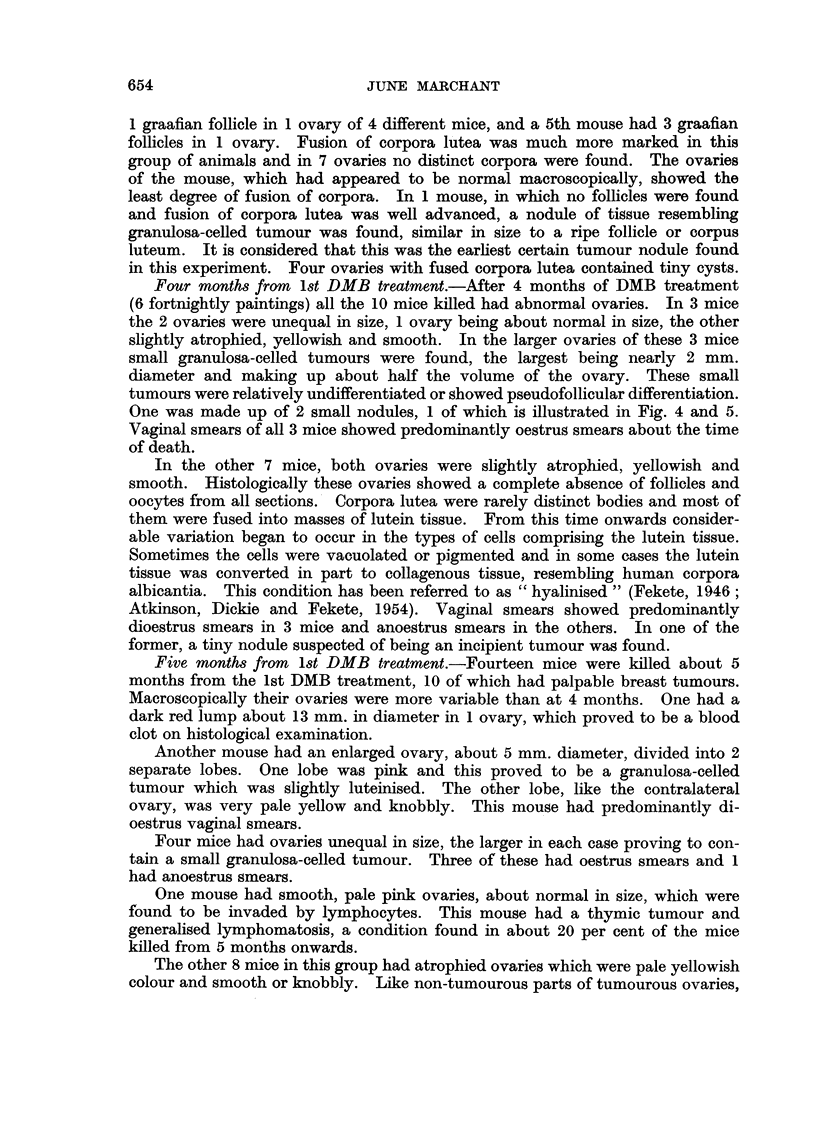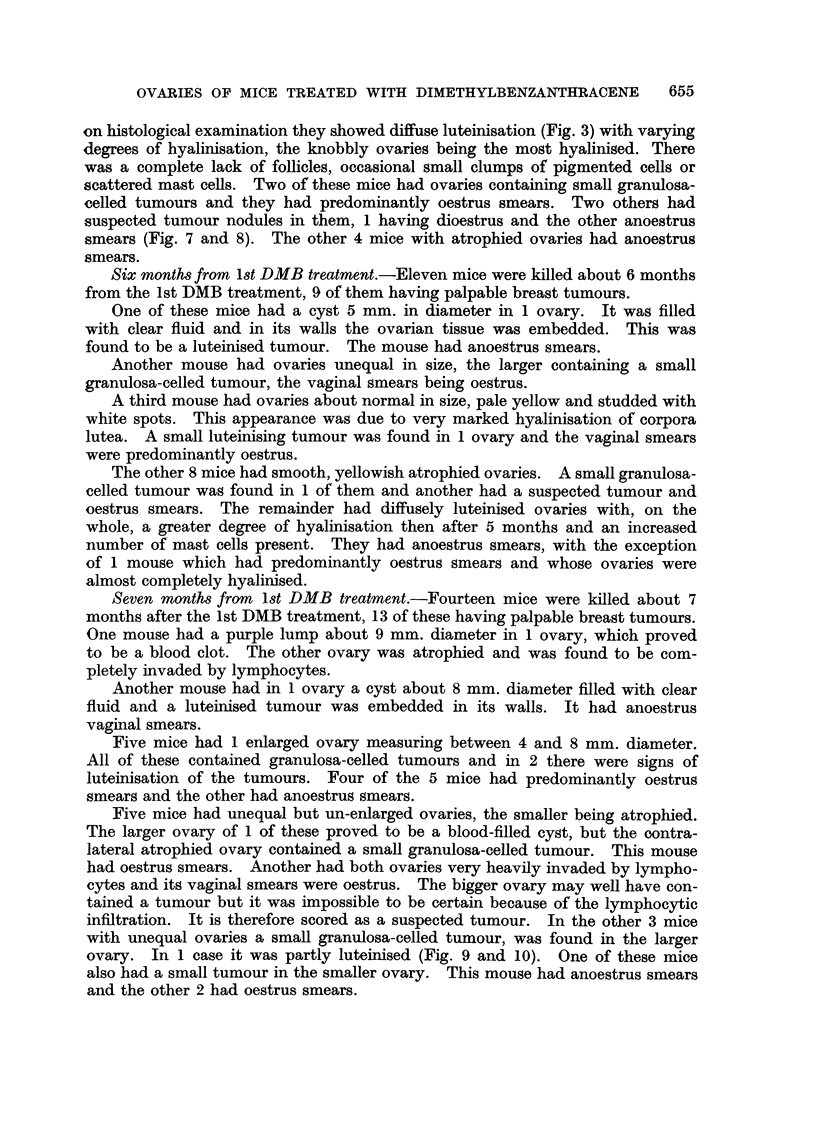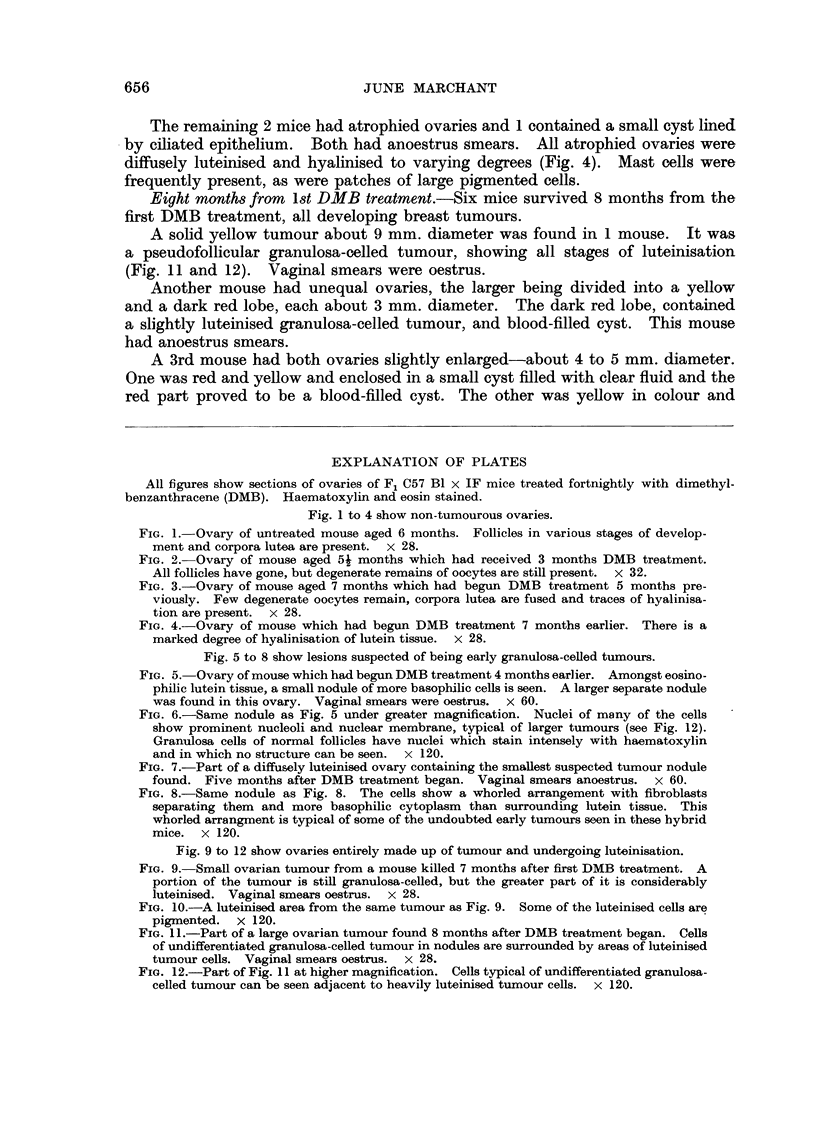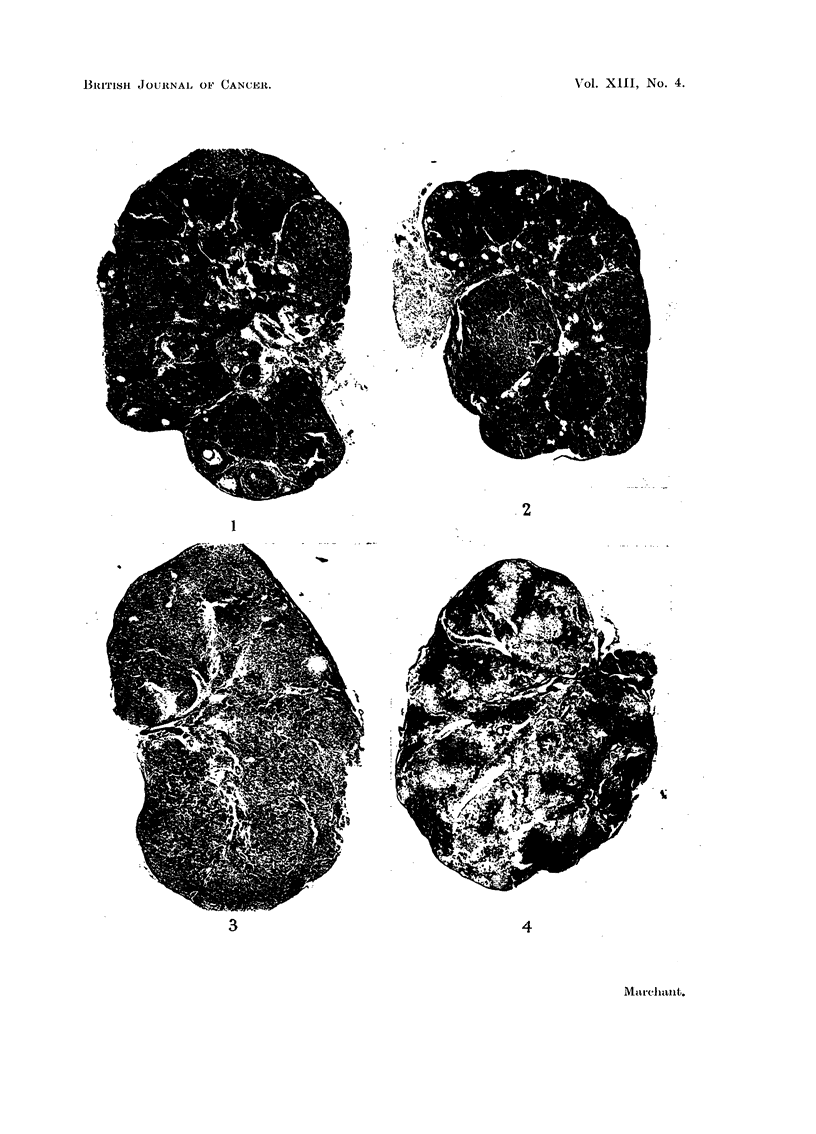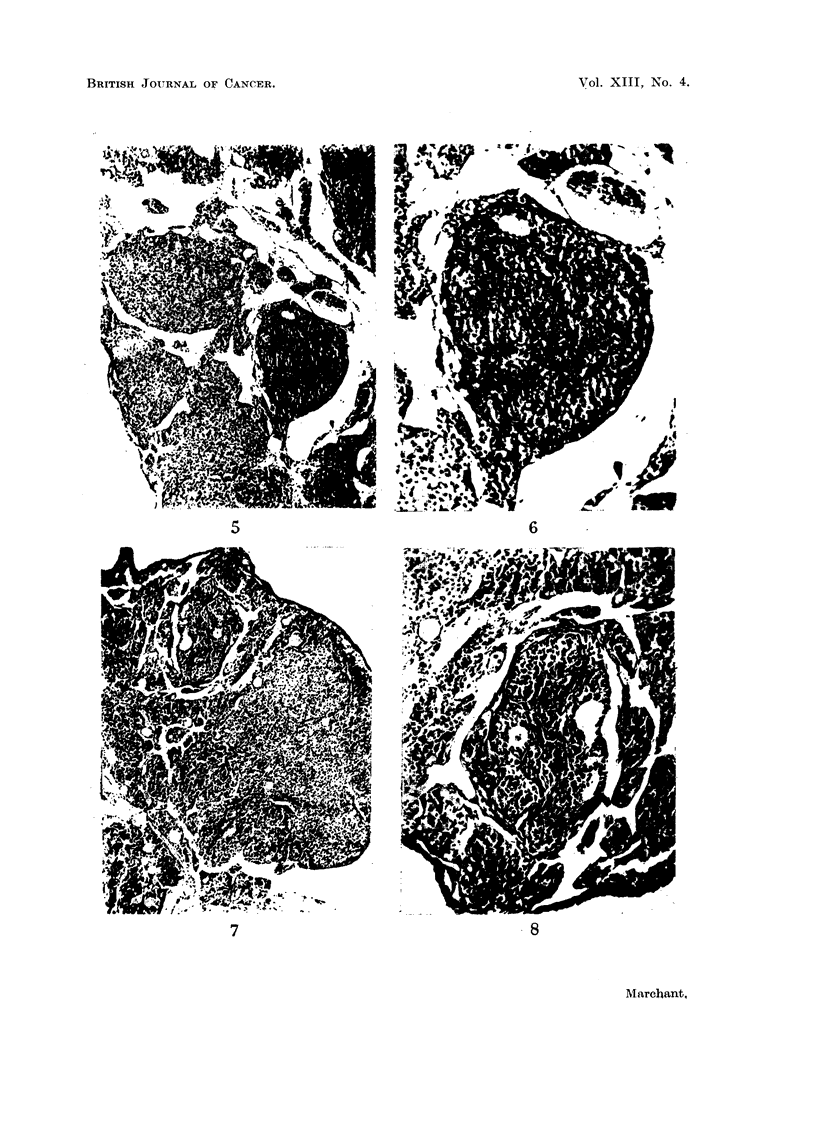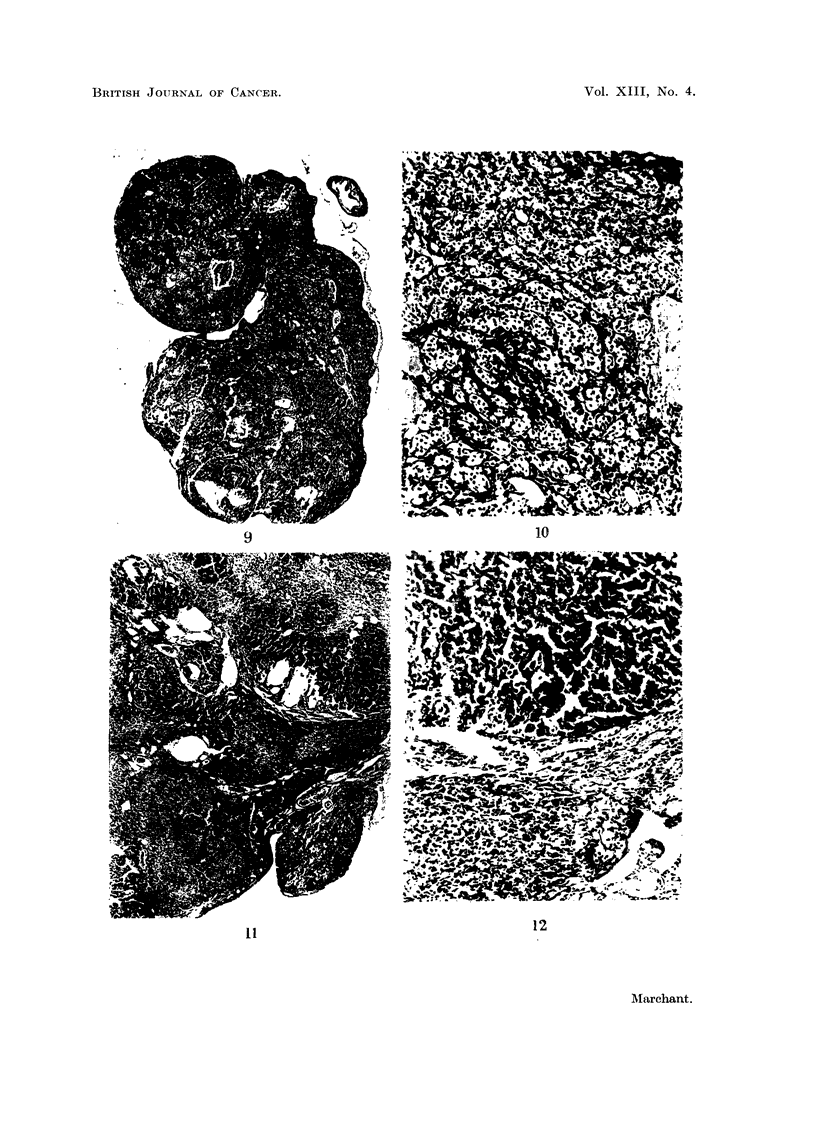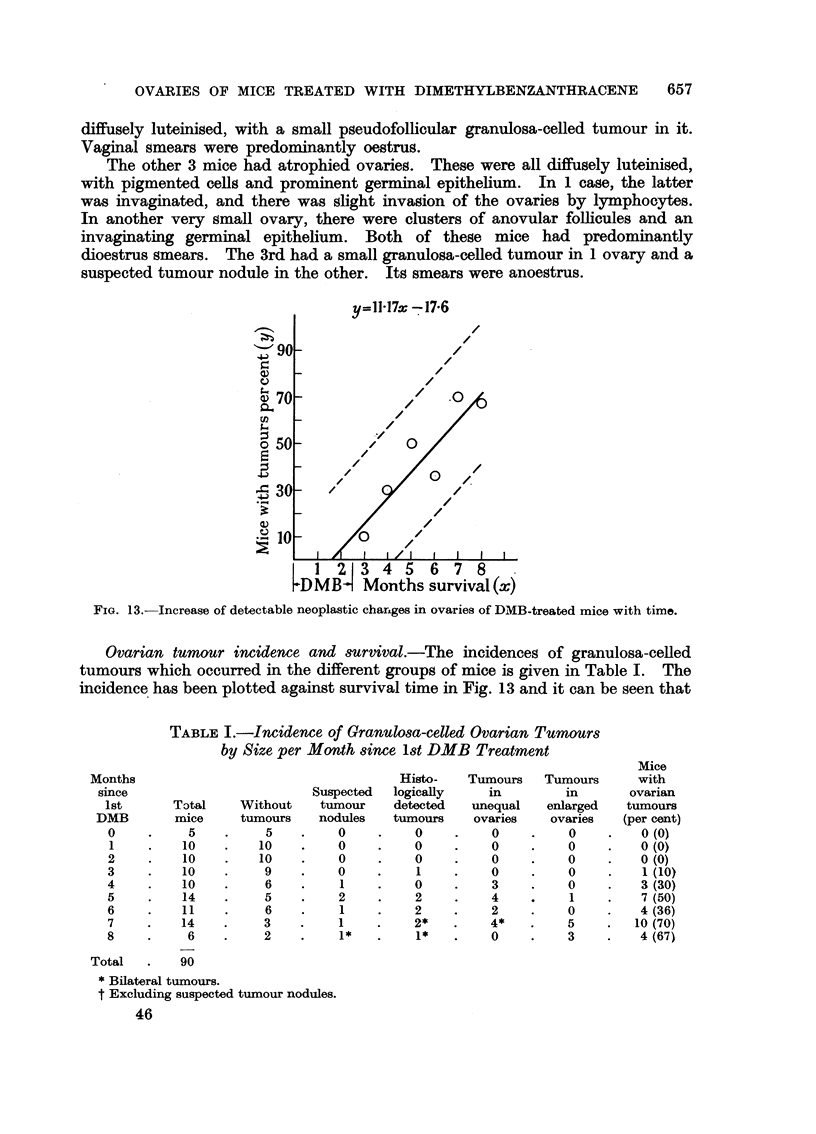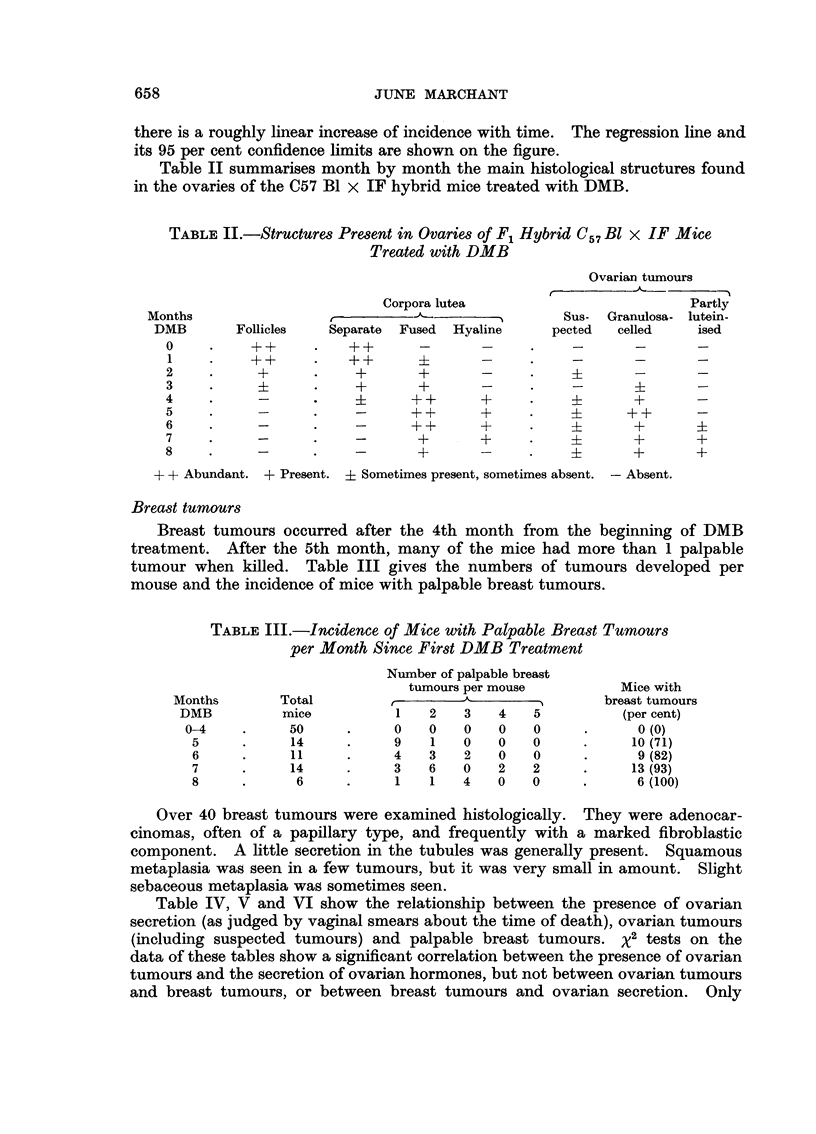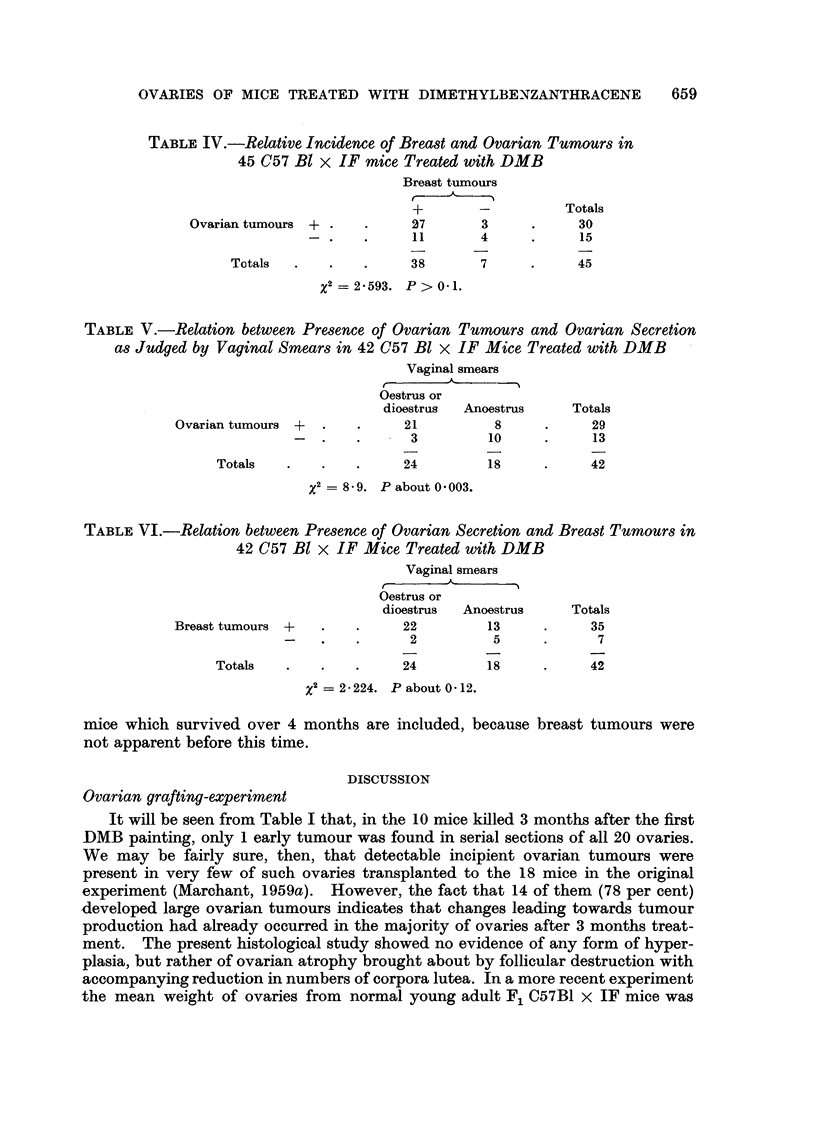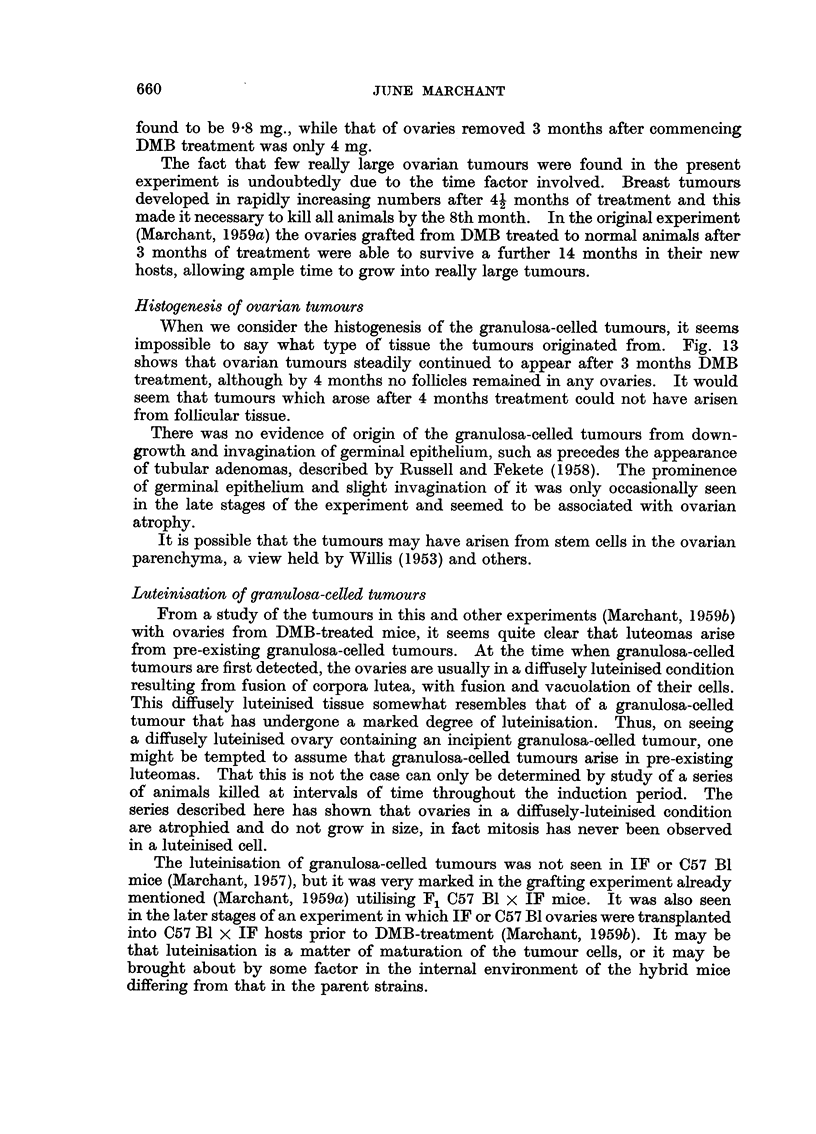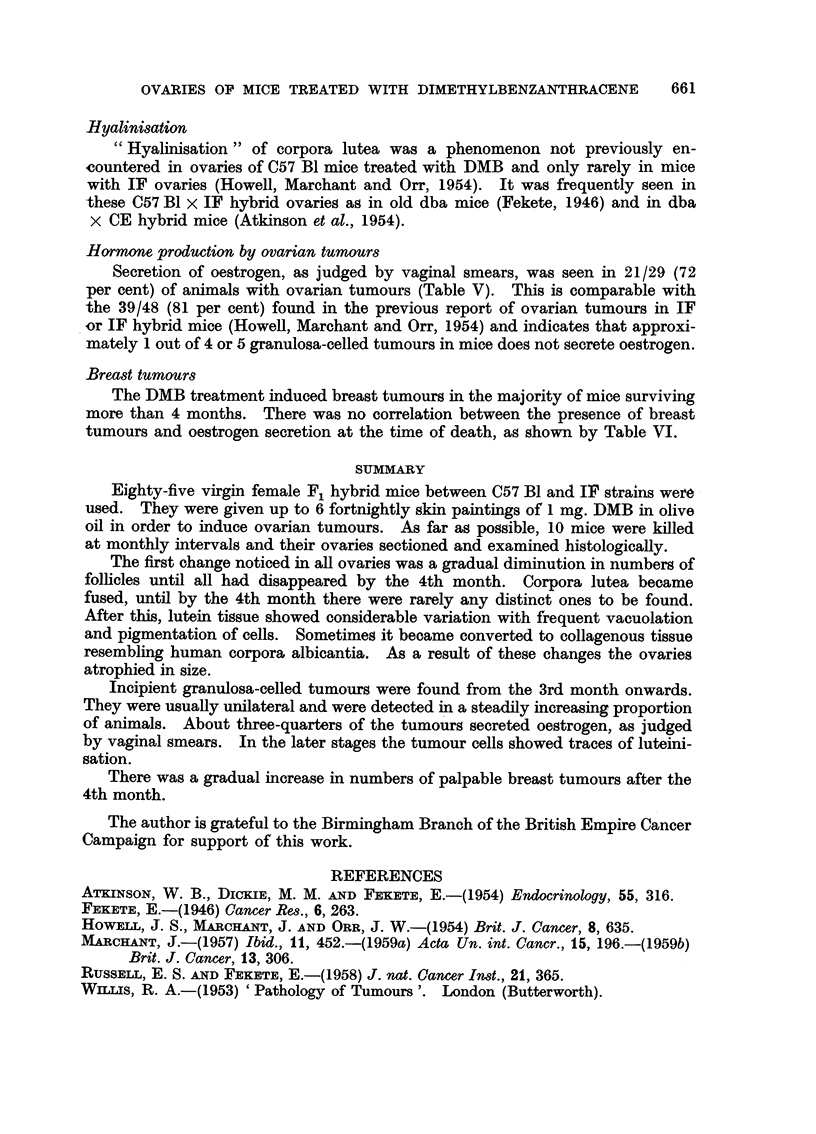# Changes in the Ovaries of Mice Treated with Dimethyl-Benzanthracene and Observations on the Subsequent Development of Tumours in Ovaries and Breasts

**DOI:** 10.1038/bjc.1959.72

**Published:** 1959-12

**Authors:** June Marchant

## Abstract

**Images:**


### CHANGES IN THE OVARIES OF MICE TREATED WITH DIMETHYLBENZANTHRACENE AND OBSERVATIONS ON THE SUBSEQUENT DEVELOPMENT OF TUMOURS IN OVARIES AND BREASTS

JUNE MARCHANT

*From the Cancer Research Laboratories, Medical School, Birmingham,* 15

Received for publication September 3, 1959

It has previously been shown that fortnightly skin paintings of an oily solution of dimethylbenzanthracene (DMB) induce a high incidence of granulosa-celled tumours of the ovary, as well as of breast tumours, in mice of the IF strain and its first generation hybrids (Howell, Marchant and Orr, 1954 ; Marchant, 1957). The earliest ovarian tumour was encountered after 4 months of treatment.

In a subsequent experiment ovaries from $F_1$ C57 Bl × IF mice, which had been treated with DMB for 3 months, were transplanted bilaterally to the ovarian capsules of untreated mice. In 14 out of 18 of these animals large ovarian tumours were found 15 months later, many of them being luteinised (Marchant, 1959*a*). At the same time a preliminary account was given of the incidence of ovarian tumour nodules found in a series of DMB-treated mice killed at monthly intervals after beginning the treatment, for it was important to know whether or not incipient tumours were already present in the transplanted ovaries.

The present report gives further details of this attempt to discover at what stage in DMB treatment tumour nodules first appeared in the ovaries. It describes the histological changes that preceded the appearance of granulosa-celled tumours, as well as those that occurred simultaneously in non-tumourous ovaries. It also gives observations on the luteinisation of established granulosa-celled tumours, which was not seen in mice of the IF strain or C57 Bl strain reported previously (Marchant, 1957). Finally, it reports the incidence and histology of the palpable breast tumours which appeared after the 4th month of the experiment.

### MATERIALS AND METHOD

Eighty-five virgin female mice lacking the mammary tumour agent were used in this experiment. They were $F_1$ hybrids derived from C57 Bl mothers and IF fathers. They were housed 5 in a box and fed on rat cubes known as the Thompson diet.

When the mice were between $2\frac{1}{2}$ and 4 months old, fortnightly skin paintings of 0·5 per cent DMB in olive oil were commenced. Up to 6 treatments were given, each mouse receiving about 0·2 ml. (1 mg.) distributed in 16 spots over dorsal and ventral surfaces at each painting.

Ten mice were killed at monthly intervals at the beginning of the experiment. After the 4th month breast tumours made their appearance, growing rapidly and necessitating the killing of some animals at irregular times. On account of this, the results reported below include some mice which had to be killed within 2 weeks of the specified month.

For a short while before death, vaginal smears were taken from nearly all the mice surviving 4 months or more, to determine whether the ovaries were secreting oestrogen or not.

At autopsy the mice were examined for palpable breast tumours, many of which were removed for histological examination. All ovaries were examined and removed. Those from mice up to and including the 5th month of treatment were serially sectioned. After that time representative sections from several different levels were mounted. Sections were stained with haematoxylin and eosin.

The ovaries of 5 untreated mice aged 6 months were also examined and serially sectioned.

In the early stages follicles and corpora lutea in the largest sections from each ovary were counted under low power to give a rough quantitative estimate of changes.

### RESULTS

### *Ovaries*

*Untreated mice.*—The ovaries of the 5 untreated mice, which were 6 months old, were about 3 × 4 mm. in diameter, roughly kidney-shaped, coloured pale pink and studded with bright pink, white or pale yellow spots.

In histological sections, numerous follicles and corpora lutea were seen, bounded by a stroma of fibrous and vascular tissue (Fig. 1). The follicles occasionally contained 2 oocytes. They, together with the corpora lutea, were situated towards the periphery. A few tiny clusters of lipochrome cells were seen. The largest sections from each of the 10 ovaries were examined in detail. They contained from 12 to 22 follicles with granulosa-cells (mean 15·8) and 13–27 fragments of corpora lutea (mean 19·4) per section.

*One month DMB treatment.*—After 1 month of DMB treatment (2 fortnightly paintings) the ovaries of the 10 mice examined appeared similar to those of untreated animals.

Histologically, too, there was little difference from normal. The largest sections, however, contained slightly fewer follicles and corpora lutea. The follicles numbered 5–19 (mean 12·7) and the corpora lutea 9–24 (mean 17·5). In 4 of the animals a few of the corpora lutea appeared to be merging.

*Two months DMB treatment.*—After 2 months of DMB treatment the ovaries of the 10 mice killed appeared macroscopically normal. In histological sections, a slight increase in numbers of merging corpora lutea was seen and the numbers of follicles counted in the largest sections of each ovary had fallen considerably. The follicles numbered 0–8 (mean 3) and the numbers of individual corpora lutea were 8–23 (mean 15·5).

*Three months DMB treatment.*—After 3 months of DMB treatment (6 fortnightly paintings) only 1 mouse of the 10 killed had what appeared to be normal ovaries macroscopically. The other ovaries in the group were atrophied, fairly smooth and yellowish.

All 20 ovaries were very abnormal histologically (Fig. 2). In the largest sections no follicles were found, but examination of all the serial sections revealed

1 graafian follicle in 1 ovary of 4 different mice, and a 5th mouse had 3 graafian follicles in 1 ovary. Fusion of corpora lutea was much more marked in this group of animals and in 7 ovaries no distinct corpora were found. The ovaries of the mouse, which had appeared to be normal macroscopically, showed the least degree of fusion of corpora. In 1 mouse, in which no follicles were found and fusion of corpora lutea was well advanced, a nodule of tissue resembling granulosa-celled tumour was found, similar in size to a ripe follicle or corpus luteum. It is considered that this was the earliest certain tumour nodule found in this experiment. Four ovaries with fused corpora lutea contained tiny cysts.

*Four months from 1st DMB treatment.*—After 4 months of DMB treatment (6 fortnightly paintings) all the 10 mice killed had abnormal ovaries. In 3 mice the 2 ovaries were unequal in size, 1 ovary being about normal in size, the other slightly atrophied, yellowish and smooth. In the larger ovaries of these 3 mice small granulosa-celled tumours were found, the largest being nearly 2 mm. diameter and making up about half the volume of the ovary. These small tumours were relatively undifferentiated or showed pseudofollicular differentiation. One was made up of 2 small nodules, 1 of which is illustrated in Fig. 4 and 5. Vaginal smears of all 3 mice showed predominantly oestrus smears about the time of death.

In the other 7 mice, both ovaries were slightly atrophied, yellowish and smooth. Histologically these ovaries showed a complete absence of follicles and oocytes from all sections. Corpora lutea were rarely distinct bodies and most of them were fused into masses of lutein tissue. From this time onwards considerable variation began to occur in the types of cells comprising the lutein tissue. Sometimes the cells were vacuolated or pigmented and in some cases the lutein tissue was converted in part to collagenous tissue, resembling human corpora albicantia. This condition has been referred to as " hyalinised " (Fekete, 1946 ; Atkinson, Dickie and Fekete, 1954). Vaginal smears showed predominantly dioestrus smears in 3 mice and anoestrus smears in the others. In one of the former, a tiny nodule suspected of being an incipient tumour was found.

*Five months from 1st DMB treatment.*—Fourteen mice were killed about 5 months from the 1st DMB treatment, 10 of which had palpable breast tumours. Macroscopically their ovaries were more variable than at 4 months. One had a dark red lump about 13 mm. in diameter in 1 ovary, which proved to be a blood clot on histological examination.

Another mouse had an enlarged ovary, about 5 mm. diameter, divided into 2 separate lobes. One lobe was pink and this proved to be a granulosa-celled tumour which was slightly luteinised. The other lobe, like the contralateral ovary, was very pale yellow and knobbly. This mouse had predominantly dioestrus vaginal smears.

Four mice had ovaries unequal in size, the larger in each case proving to contain a small granulosa-celled tumour. Three of these had oestrus smears and 1 had anoestrus smears.

One mouse had smooth, pale pink ovaries, about normal in size, which were found to be invaded by lymphocytes. This mouse had a thymic tumour and generalised lymphomatosis, a condition found in about 20 per cent of the mice killed from 5 months onwards.

The other 8 mice in this group had atrophied ovaries which were pale yellowish colour and smooth or knobbly. Like non-tumourous parts of tumourous ovaries, on histological examination they showed diffuse luteinisation (Fig. 3) with varying degrees of hyalinisation, the knobbly ovaries being the most hyalinised. There was a complete lack of follicles, occasional small clumps of pigmented cells or scattered mast cells. Two of these mice had ovaries containing small granulosa-celled tumours and they had predominantly oestrus smears. Two others had suspected tumour nodules in them, 1 having dioestrus and the other anoestrus smears (Fig. 7 and 8). The other 4 mice with atrophied ovaries had anoestrus smears.

*Six months from 1st DMB treatment.*—Eleven mice were killed about 6 months from the 1st DMB treatment, 9 of them having palpable breast tumours.

One of these mice had a cyst 5 mm. in diameter in 1 ovary. It was filled with clear fluid and in its walls the ovarian tissue was embedded. This was found to be a luteinised tumour. The mouse had anoestrus smears.

Another mouse had ovaries unequal in size, the larger containing a small granulosa-celled tumour, the vaginal smears being oestrus.

A third mouse had ovaries about normal in size, pale yellow and studded with white spots. This appearance was due to very marked hyalinisation of corpora lutea. A small luteinising tumour was found in 1 ovary and the vaginal smears were predominantly oestrus.

The other 8 mice had smooth, yellowish atrophied ovaries. A small granulosa-celled tumour was found in 1 of them and another had a suspected tumour and oestrus smears. The remainder had diffusely luteinised ovaries with, on the whole, a greater degree of hyalinisation then after 5 months and an increased number of mast cells present. They had anoestrus smears, with the exception of 1 mouse which had predominantly oestrus smears and whose ovaries were almost completely hyalinised.

*Seven months from 1st DMB treatment.*—Fourteen mice were killed about 7 months after the 1st DMB treatment, 13 of these having palpable breast tumours. One mouse had a purple lump about 9 mm. diameter in 1 ovary, which proved to be a blood clot. The other ovary was atrophied and was found to be completely invaded by lymphocytes.

Another mouse had in 1 ovary a cyst about 8 mm. diameter filled with clear fluid and a luteinised tumour was embedded in its walls. It had anoestrus vaginal smears.

Five mice had 1 enlarged ovary measuring between 4 and 8 mm. diameter. All of these contained granulosa-celled tumours and in 2 there were signs of luteinisation of the tumours. Four of the 5 mice had predominantly oestrus smears and the other had anoestrus smears.

Five mice had unequal but un-enlarged ovaries, the smaller being atrophied. The larger ovary of 1 of these proved to be a blood-filled cyst, but the contralateral atrophied ovary contained a small granulosa-celled tumour. This mouse had oestrus smears. Another had both ovaries very heavily invaded by lymphocytes and its vaginal smears were oestrus. The bigger ovary may well have contained a tumour but it was impossible to be certain because of the lymphocytic infiltration. It is therefore scored as a suspected tumour. In the other 3 mice with unequal ovaries a small granulosa-celled tumour, was found in the larger ovary. In 1 case it was partly luteinised (Fig. 9 and 10). One of these mice also had a small tumour in the smaller ovary. This mouse had anoestrus smears and the other 2 had oestrus smears.

The remaining 2 mice had atrophied ovaries and 1 contained a small cyst lined by ciliated epithelium. Both had anoestrus smears. All atrophied ovaries were diffusely luteinised and hyalinised to varying degrees (Fig. 4). Mast cells were frequently present, as were patches of large pigmented cells.

*Eight months from 1st DMB treatment.*—Six mice survived 8 months from the first DMB treatment, all developing breast tumours.

A solid yellow tumour about 9 mm. diameter was found in 1 mouse. It was a pseudofollicular granulosa-celled tumour, showing all stages of luteinisation (Fig. 11 and 12). Vaginal smears were oestrus.

Another mouse had unequal ovaries, the larger being divided into a yellow and a dark red lobe, each about 3 mm. diameter. The dark red lobe, contained a slightly luteinised granulosa-celled tumour, and blood-filled cyst. This mouse had anoestrus smears.

A 3rd mouse had both ovaries slightly enlarged—about 4 to 5 mm. diameter. One was red and yellow and enclosed in a small cyst filled with clear fluid and the red part proved to be a blood-filled cyst. The other was yellow in colour and

---

### EXPLANATION OF PLATES

All figures show sections of ovaries of $F_1$ C57 Bl × IF mice treated fortnightly with dimethylbenzanthracene (DMB). Haematoxylin and eosin stained.

Fig. 1 to 4 show non-tumourous ovaries.

FIG. 1.—Ovary of untreated mouse aged 6 months. Follicles in various stages of development and corpora lutea are present. × 28.

FIG. 2.—Ovary of mouse aged 5½ months which had received 3 months DMB treatment. All follicles have gone, but degenerate remains of oocytes are still present. × 32.

FIG. 3.—Ovary of mouse aged 7 months which had begun DMB treatment 5 months previously. Few degenerate oocytes remain, corpora lutea are fused and traces of hyalinisation are present. × 28.

FIG. 4.—Ovary of mouse which had begun DMB treatment 7 months earlier. There is a marked degree of hyalinisation of lutein tissue. × 28.

Fig. 5 to 8 show lesions suspected of being early granulosa-celled tumours.

FIG. 5.—Ovary of mouse which had begun DMB treatment 4 months earlier. Amongst eosinophilic lutein tissue, a small nodule of more basophilic cells is seen. A larger separate nodule was found in this ovary. Vaginal smears were oestrus. × 60.

FIG. 6.—Same nodule as Fig. 5 under greater magnification. Nuclei of many of the cells show prominent nucleoli and nuclear membrane, typical of larger tumours (see Fig. 12). Granulosa cells of normal follicles have nuclei which stain intensely with haematoxylin and in which no structure can be seen. × 120.

FIG. 7.—Part of a diffusely luteinised ovary containing the smallest suspected tumour nodule found. Five months after DMB treatment began. Vaginal smears anoestrus. × 60.

FIG. 8.—Same nodule as Fig. 8. The cells show a whorled arrangement with fibroblasts separating them and more basophilic cytoplasm than surrounding lutein tissue. This whorled arrangment is typical of some of the undoubted early tumours seen in these hybrid mice. × 120.

Fig. 9 to 12 show ovaries entirely made up of tumour and undergoing luteinisation.

FIG. 9.—Small ovarian tumour from a mouse killed 7 months after first DMB treatment. A portion of the tumour is still granulosa-celled, but the greater part of it is considerably luteinised. Vaginal smears oestrus. × 28.

FIG. 10.—A luteinised area from the same tumour as Fig. 9. Some of the luteinised cells are pigmented. × 120.

FIG. 11.—Part of a large ovarian tumour found 8 months after DMB treatment began. Cells of undifferentiated granulosa-celled tumour in nodules are surrounded by areas of luteinised tumour cells. Vaginal smears oestrus. × 28.

FIG. 12.—Part of Fig. 11 at higher magnification. Cells typical of undifferentiated granulosa-celled tumour can be seen adjacent to heavily luteinised tumour cells. × 120.

1

2

3

4

Marchant.

5

6

7

8

9

10

11

12

Marchant.

diffusely luteinised, with a small pseudofollicular granulosa-celled tumour in it. Vaginal smears were predominantly oestrus.

The other 3 mice had atrophied ovaries. These were all diffusely luteinised, with pigmented cells and prominent germinal epithelium. In 1 case, the latter was invaginated, and there was slight invasion of the ovaries by lymphocytes. In another very small ovary, there were clusters of anovular follicules and an invaginating germinal epithelium. Both of these mice had predominantly dioestrus smears. The 3rd had a small granulosa-celled tumour in 1 ovary and a suspected tumour nodule in the other. Its smears were anoestrus.

FIG. 13.—Increase of detectable neoplastic changes in ovaries of DMB-treated mice with time.

*Ovarian tumour incidence and survival.*—The incidences of granulosa-celled tumours which occurred in the different groups of mice is given in Table I. The incidence has been plotted against survival time in Fig. 13 and it can be seen that

TABLE I.—*Incidence of Granulosa-celled Ovarian Tumours by Size per Month since 1st DMB Treatment*

| Months since 1st DMB | Total mice | Without tumours | Suspected tumour nodules | Histo-logically detected tumours | Tumours in unequal ovaries | Tumours in enlarged ovaries | Mice with ovarian tumours (per cent) |
|---|---|---|---|---|---|---|---|
| 0 | 5 | 5 | 0 | 0 | 0 | 0 | 0 (0) |
| 1 | 10 | 10 | 0 | 0 | 0 | 0 | 0 (0) |
| 2 | 10 | 10 | 0 | 0 | 0 | 0 | 0 (0) |
| 3 | 10 | 9 | 0 | 1 | 0 | 0 | 1 (10) |
| 4 | 10 | 6 | 1 | 0 | 3 | 0 | 3 (30) |
| 5 | 14 | 5 | 2 | 2 | 4 | 1 | 7 (50) |
| 6 | 11 | 6 | 1 | 2 | 2 | 0 | 4 (36) |
| 7 | 14 | 3 | 1 | 2* | 4* | 5 | 10 (70) |
| 8 | 6 | 2 | 1* | 1* | 0 | 3 | 4 (67) |
| Total | 90 | | | | | | |

* Bilateral tumours.
† Excluding suspected tumour nodules.

there is a roughly linear increase of incidence with time. The regression line and its 95 per cent confidence limits are shown on the figure.

Table II summarises month by month the main histological structures found in the ovaries of the C57 Bl × IF hybrid mice treated with DMB.

TABLE II.—*Structures Present in Ovaries of $F_1$ Hybrid $C_{57}Bl$ × IF Mice Treated with DMB*

| Months DMB | Follicles | Corpora lutea: Separate | Corpora lutea: Fused | Corpora lutea: Hyaline | Ovarian tumours: Suspected | Ovarian tumours: Granulosa-celled | Ovarian tumours: Partly luteinised |
|---|---|---|---|---|---|---|---|
| 0 | ++ | ++ | – | – | – | – | – |
| 1 | ++ | ++ | ± | – | – | – | – |
| 2 | + | + | + | – | ± | – | – |
| 3 | ± | + | + | – | – | ± | – |
| 4 | – | ± | ++ | + | ± | + | – |
| 5 | – | – | ++ | + | ± | ++ | – |
| 6 | – | – | ++ | + | ± | + | ± |
| 7 | – | – | + | + | ± | + | + |
| 8 | – | – | + | – | ± | + | + |

++ Abundant. + Present. ± Sometimes present, sometimes absent. – Absent.

*Breast tumours*

Breast tumours occurred after the 4th month from the beginning of DMB treatment. After the 5th month, many of the mice had more than 1 palpable tumour when killed. Table III gives the numbers of tumours developed per mouse and the incidence of mice with palpable breast tumours.

TABLE III.—*Incidence of Mice with Palpable Breast Tumours per Month Since First DMB Treatment*

| Months DMB | Total mice | Number of palpable breast tumours per mouse: 1 | 2 | 3 | 4 | 5 | Mice with breast tumours (per cent) |
|---|---|---|---|---|---|---|---|
| 0–4 | 50 | 0 | 0 | 0 | 0 | 0 | 0 (0) |
| 5 | 14 | 9 | 1 | 0 | 0 | 0 | 10 (71) |
| 6 | 11 | 4 | 3 | 2 | 0 | 0 | 9 (82) |
| 7 | 14 | 3 | 6 | 0 | 2 | 2 | 13 (93) |
| 8 | 6 | 1 | 1 | 4 | 0 | 0 | 6 (100) |

Over 40 breast tumours were examined histologically. They were adenocarcinomas, often of a papillary type, and frequently with a marked fibroblastic component. A little secretion in the tubules was generally present. Squamous metaplasia was seen in a few tumours, but it was very small in amount. Slight sebaceous metaplasia was sometimes seen.

Table IV, V and VI show the relationship between the presence of ovarian secretion (as judged by vaginal smears about the time of death), ovarian tumours (including suspected tumours) and palpable breast tumours. $\chi^2$ tests on the data of these tables show a significant correlation between the presence of ovarian tumours and the secretion of ovarian hormones, but not between ovarian tumours and breast tumours, or between breast tumours and ovarian secretion. Only

TABLE IV.—*Relative Incidence of Breast and Ovarian Tumours in 45 C57 Bl × IF mice Treated with DMB*

| | | Breast tumours + | Breast tumours − | Totals |
|---|---|---|---|---|
| Ovarian tumours | + | 27 | 3 | 30 |
| | − | 11 | 4 | 15 |
| Totals | | 38 | 7 | 45 |

$\chi^2 = 2\cdot593$. $P > 0\cdot1$.

TABLE V.—*Relation between Presence of Ovarian Tumours and Ovarian Secretion as Judged by Vaginal Smears in 42 C57 Bl × IF Mice Treated with DMB*

| | | Vaginal smears: Oestrus or dioestrus | Vaginal smears: Anoestrus | Totals |
|---|---|---|---|---|
| Ovarian tumours | + | 21 | 8 | 29 |
| | − | 3 | 10 | 13 |
| Totals | | 24 | 18 | 42 |

$\chi^2 = 8\cdot9$. $P$ about $0\cdot003$.

TABLE VI.—*Relation between Presence of Ovarian Secretion and Breast Tumours in 42 C57 Bl × IF Mice Treated with DMB*

| | | Vaginal smears: Oestrus or dioestrus | Vaginal smears: Anoestrus | Totals |
|---|---|---|---|---|
| Breast tumours | + | 22 | 13 | 35 |
| | − | 2 | 5 | 7 |
| Totals | | 24 | 18 | 42 |

$\chi^2 = 2\cdot224$. $P$ about $0\cdot12$.

mice which survived over 4 months are included, because breast tumours were not apparent before this time.

### DISCUSSION

*Ovarian grafting-experiment*

It will be seen from Table I that, in the 10 mice killed 3 months after the first DMB painting, only 1 early tumour was found in serial sections of all 20 ovaries. We may be fairly sure, then, that detectable incipient ovarian tumours were present in very few of such ovaries transplanted to the 18 mice in the original experiment (Marchant, 1959*a*). However, the fact that 14 of them (78 per cent) developed large ovarian tumours indicates that changes leading towards tumour production had already occurred in the majority of ovaries after 3 months treatment. The present histological study showed no evidence of any form of hyperplasia, but rather of ovarian atrophy brought about by follicular destruction with accompanying reduction in numbers of corpora lutea. In a more recent experiment the mean weight of ovaries from normal young adult $F_1$ C57Bl × IF mice was found to be 9·8 mg., while that of ovaries removed 3 months after commencing DMB treatment was only 4 mg.

The fact that few really large ovarian tumours were found in the present experiment is undoubtedly due to the time factor involved. Breast tumours developed in rapidly increasing numbers after 4½ months of treatment and this made it necessary to kill all animals by the 8th month. In the original experiment (Marchant, 1959*a*) the ovaries grafted from DMB treated to normal animals after 3 months of treatment were able to survive a further 14 months in their new hosts, allowing ample time to grow into really large tumours.

*Histogenesis of ovarian tumours*

When we consider the histogenesis of the granulosa-celled tumours, it seems impossible to say what type of tissue the tumours originated from. Fig. 13 shows that ovarian tumours steadily continued to appear after 3 months DMB treatment, although by 4 months no follicles remained in any ovaries. It would seem that tumours which arose after 4 months treatment could not have arisen from follicular tissue.

There was no evidence of origin of the granulosa-celled tumours from down-growth and invagination of germinal epithelium, such as precedes the appearance of tubular adenomas, described by Russell and Fekete (1958). The prominence of germinal epithelium and slight invagination of it was only occasionally seen in the late stages of the experiment and seemed to be associated with ovarian atrophy.

It is possible that the tumours may have arisen from stem cells in the ovarian parenchyma, a view held by Willis (1953) and others.

*Luteinisation of granulosa-celled tumours*

From a study of the tumours in this and other experiments (Marchant, 1959*b*) with ovaries from DMB-treated mice, it seems quite clear that luteomas arise from pre-existing granulosa-celled tumours. At the time when granulosa-celled tumours are first detected, the ovaries are usually in a diffusely luteinised condition resulting from fusion of corpora lutea, with fusion and vacuolation of their cells. This diffusely luteinised tissue somewhat resembles that of a granulosa-celled tumour that has undergone a marked degree of luteinisation. Thus, on seeing a diffusely luteinised ovary containing an incipient granulosa-celled tumour, one might be tempted to assume that granulosa-celled tumours arise in pre-existing luteomas. That this is not the case can only be determined by study of a series of animals killed at intervals of time throughout the induction period. The series described here has shown that ovaries in a diffusely-luteinised condition are atrophied and do not grow in size, in fact mitosis has never been observed in a luteinised cell.

The luteinisation of granulosa-celled tumours was not seen in IF or C57 Bl mice (Marchant, 1957), but it was very marked in the grafting experiment already mentioned (Marchant, 1959*a*) utilising $F_1$ C57 Bl × IF mice. It was also seen in the later stages of an experiment in which IF or C57 Bl ovaries were transplanted into C57 Bl × IF hosts prior to DMB-treatment (Marchant, 1959*b*). It may be that luteinisation is a matter of maturation of the tumour cells, or it may be brought about by some factor in the internal environment of the hybrid mice differing from that in the parent strains.

*Hyalinisation*

"Hyalinisation" of corpora lutea was a phenomenon not previously encountered in ovaries of C57 Bl mice treated with DMB and only rarely in mice with IF ovaries (Howell, Marchant and Orr, 1954). It was frequently seen in these C57 Bl × IF hybrid ovaries as in old dba mice (Fekete, 1946) and in dba × CE hybrid mice (Atkinson *et al.*, 1954).

*Hormone production by ovarian tumours*

Secretion of oestrogen, as judged by vaginal smears, was seen in 21/29 (72 per cent) of animals with ovarian tumours (Table V). This is comparable with the 39/48 (81 per cent) found in the previous report of ovarian tumours in IF or IF hybrid mice (Howell, Marchant and Orr, 1954) and indicates that approximately 1 out of 4 or 5 granulosa-celled tumours in mice does not secrete oestrogen.

*Breast tumours*

The DMB treatment induced breast tumours in the majority of mice surviving more than 4 months. There was no correlation between the presence of breast tumours and oestrogen secretion at the time of death, as shown by Table VI.

### SUMMARY

Eighty-five virgin female $F_1$ hybrid mice between C57 Bl and IF strains were used. They were given up to 6 fortnightly skin paintings of 1 mg. DMB in olive oil in order to induce ovarian tumours. As far as possible, 10 mice were killed at monthly intervals and their ovaries sectioned and examined histologically.

The first change noticed in all ovaries was a gradual diminution in numbers of follicles until all had disappeared by the 4th month. Corpora lutea became fused, until by the 4th month there were rarely any distinct ones to be found. After this, lutein tissue showed considerable variation with frequent vacuolation and pigmentation of cells. Sometimes it became converted to collagenous tissue resembling human corpora albicantia. As a result of these changes the ovaries atrophied in size.

Incipient granulosa-celled tumours were found from the 3rd month onwards. They were usually unilateral and were detected in a steadily increasing proportion of animals. About three-quarters of the tumours secreted oestrogen, as judged by vaginal smears. In the later stages the tumour cells showed traces of luteinisation.

There was a gradual increase in numbers of palpable breast tumours after the 4th month.

The author is grateful to the Birmingham Branch of the British Empire Cancer Campaign for support of this work.

### REFERENCES

Atkinson, W. B., Dickie, M. M. and Fekete, E.—(1954) *Endocrinology*, **55**, 316.
Fekete, E.—(1946) *Cancer Res.*, **6**, 263.
Howell, J. S., Marchant, J. and Orr, J. W.—(1954) *Brit. J. Cancer*, **8**, 635.
Marchant, J.—(1957) *Ibid.*, **11**, 452.—(1959*a*) *Acta Un. int. Cancr.*, **15**, 196.—(1959*b*) *Brit. J. Cancer*, **13**, 306.
Russell, E. S. and Fekete, E.—(1958) *J. nat. Cancer Inst.*, **21**, 365.
Willis, R. A.—(1953) 'Pathology of Tumours'. London (Butterworth).